# (4*Z*)-4-[(4-Chloro­anilino)(phen­yl)methyl­ene]-3-methyl-1-phenyl-1*H*-pyrazol-5(4*H*)-one

**DOI:** 10.1107/S1600536809054816

**Published:** 2009-12-24

**Authors:** Xiaodong Chi, Jing Xiao, Yuhong Yin, Min Xia

**Affiliations:** a Department of Chemistry, Zhejiang Sci-Tech University, Hangzhou 310018, People’s Republic of China

## Abstract

The title compound, C_23_H_18_ClN_3_O, was synthesized by the reaction of 4-chloro­aniline and 4-benzoyl-3-methyl-1-phenyl-1*H*-pyrazol-5(4*H*)-one. The terminal benzene rings are twisted at dihedral angles of 48.3 (2), 71.4 (2) and 36.1 (2)° with respect to the central eight-atom methyl­pyrazolone/amino­methyl­ene unit. An intra­molecular N—H⋯O hydrogen bond stabilizes the planar conformation [mean deviation = 0.0398 (5) Å] of the central unit, generating an *S*(6) ring motif. The crystal packing is stabilized by van der Waals forces.

## Related literature

For the properties of β-enamino­ketones, see: Li *et al.* (2000 ▶); Zhang *et al.* (2003 ▶, 2008 ▶); Cingolani *et al.* (2006 ▶); Marchetti *et al.* (2005 ▶). For the preparation of β-enamino­ketones, see: Yang *et al.* (2004 ▶). For graph-set notation, see: Bernstein *et al.* (1995 ▶).
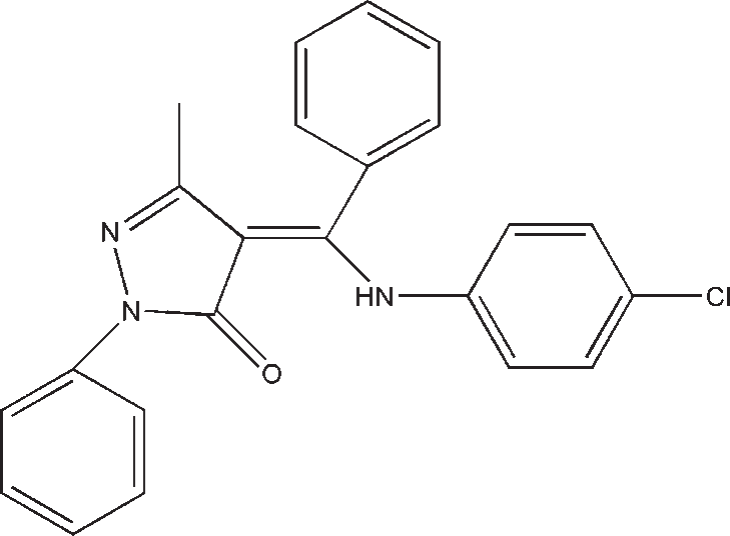


## Experimental

### 

#### Crystal data

C_23_H_18_ClN_3_O
*M* *_r_* = 387.85
Triclinic, 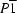

*a* = 7.4305 (15) Å
*b* = 11.069 (2) Å
*c* = 13.518 (3) Å
α = 109.28 (3)°
β = 98.78 (3)°
γ = 105.08 (3)°
*V* = 978.0 (5) Å^3^
*Z* = 2
Mo *K*α radiation
μ = 0.21 mm^−1^
*T* = 293 K
0.34 × 0.31 × 0.09 mm

#### Data collection

Rigaku R-AXIS RAPID diffractometer
Absorption correction: multi-scan (*ABSCOR*; Higashi, 1995 ▶) *T*_min_ = 0.921, *T*_max_ = 0.985
7818 measured reflections
3506 independent reflections
2198 reflections with *I* > 2σ(*I*)
*R*_int_ = 0.033

#### Refinement

*R*[*F* ^2^ > 2σ(*F* ^2^)] = 0.055
*wR*(*F* ^2^) = 0.142
*S* = 1.05
3506 reflections
259 parameters
H atoms treated by a mixture of independent and constrained refinement
Δρ_max_ = 0.61 e Å^−3^
Δρ_min_ = −0.56 e Å^−3^

### 

Data collection: *RAPID-AUTO* (Rigaku, 1998 ▶); cell refinement: *RAPID-AUTO*; data reduction: *CrystalStructure* (Rigaku/MSC, 2002 ▶); program(s) used to solve structure: *SHELXS97* (Sheldrick, 2008 ▶); program(s) used to refine structure: *SHELXL97* (Sheldrick, 2008 ▶); molecular graphics: *ORTEP-3* (Farrugia, 1997 ▶); software used to prepare material for publication: *SHELXL97* and *PLATON* (Spek, 2009 ▶).

## Supplementary Material

Crystal structure: contains datablocks global, I. DOI: 10.1107/S1600536809054816/si2229sup1.cif

Structure factors: contains datablocks I. DOI: 10.1107/S1600536809054816/si2229Isup2.hkl

Additional supplementary materials:  crystallographic information; 3D view; checkCIF report

## Figures and Tables

**Table 1:** Hydrogen-bond geometry (Å, °)

*D*—H⋯*A*	*D*—H	H⋯*A*	*D*⋯*A*	*D*—H⋯*A*
N1—H1⋯O1	0.93 (3)	1.87 (3)	2.701 (3)	146 (2)

**Fig. 1.**
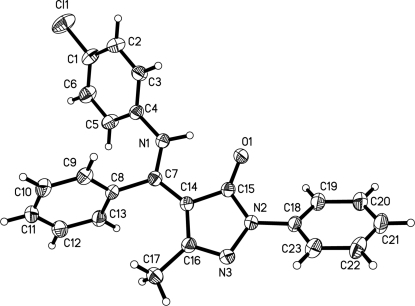
View of the molecule showing the atom-labelling scheme. Displacement ellipsoids are drawn at the 30% probability level. H atoms are represented by circles of arbitrary size.

## supplementary crystallographic information

### Comment

The 4-acyl-3-methyl-1-phenyl-1*H*-pyrazol-5(4*H*)-ones are a novel type of β-enaminoketone (Yang *et al.*, 2004) with a heterocyclic structure, which have the strong coordination to be as the extractants of trace metals, laser materials, shift reagents in NMR and so on [Marchetti *et al.*, 2005]. Apart from the similar capacity of the selective coordination with many metals [Zhang *et al.*, 2008; Cingolani *et al.*, 2006], the Schiff's base derivatives of 4-acyl-3-methyl- 1-phenyl-1*H*-pyrazol-5(4*H*)-ones have also exhibited their special photoluminescence [Zhang *et al.*, 2003] and bioactivities [Li *et al.*, 2000]. As a part of work interested in the complexes with these Schiff's bases, we herein report the preparation of the title compound and its corresponding crystal structure.

The bond lengths and angles of the title molecule (Fig. 1) are within normal ranges. The terminal benzene rings [C1–C6, C8–C13 and C18–C23] are twisted at dihedral angles of 48.3 (2), 71.4 (2) and 36.1 (2) ° with respect to the central eight atom methylpyrazolone/aminomethylene unit [mean deviation = 0.0398 (5) Å]. An intramolecular N—H···O hydrogen bond generating an S(6) ring is observed [Bernstein *et al.*, 1995]. The crystal packing is stabilized by van der Waals forces.

### Experimental

The solution of 4-chloroaniline (1.2 mmol) and 4-benzoyl-3-methyl-1-phenyl-1*H*-pyrazol- 5(4*H*)-one (1 mmol) in ethanol (10 mL) was refluxed for 5 h and the yellow precipitate was gradually formed. After cooled to the room temperature, the mixture was filtrated and the collected solid was washed with additional ethanol and dried in the air. Suitable crystals were obtained by evaporation of an ethanol/dichloromethane(1:1) mixed solution (m.p. 488–489 K).

### Refinement

The structures were solved by Direct methods and using Fourier techniques. The non-hydrogen atoms were refined anisotropically. All H-atoms were placed in idealized locations with C–H distances 0.93 - 0.96 Å and refined as riding with appropriate thermal displacement coefficients *U*_iso_(H) = 1.2 or 1.5 times *U*_eq_(bearing atom).

### Crystal data

**Table:**

C_23_H_18_ClN_3_O	*Z* = 2
*M**_r_* = 387.85	*F*(000) = 404
Triclinic, *P*1	*D*_x_ = 1.317 Mg m^−^^3^
Hall symbol: -P 1	Mo *K*α radiation, λ = 0.71073 Å
*a* = 7.4305 (15) Å	Cell parameters from 5058 reflections
*b* = 11.069 (2) Å	θ = 3.1–27.5°
*c* = 13.518 (3) Å	µ = 0.21 mm^−^^1^
α = 109.28 (3)°	*T* = 293 K
β = 98.78 (3)°	Platelet, yellow
γ = 105.08 (3)°	0.34 × 0.31 × 0.09 mm
*V* = 978.0 (5) Å^3^	

### Data collection

**Table:**

Rigaku R-AXIS RAPID diffractometer	3506 independent reflections
Radiation source: fine-focus sealed tube	2198 reflections with *I* > 2σ(*I*)
graphite	*R*_int_ = 0.033
Detector resolution: 10.00 pixels mm^-1^	θ_max_ = 25.2°, θ_min_ = 3.1°
ω scans	*h* = −8→8
Absorption correction: multi-scan (*ABSCOR*; Higashi, 1995)	*k* = −13→13
*T*_min_ = 0.921, *T*_max_ = 0.985	*l* = −16→16
7818 measured reflections	

### Refinement

**Table:**

Refinement on *F*^2^	Secondary atom site location: difference Fourier map
Least-squares matrix: full	Hydrogen site location: inferred from neighbouring sites
*R*[*F*^2^ > 2σ(*F*^2^)] = 0.055	H atoms treated by a mixture of independent and constrained refinement
*wR*(*F*^2^) = 0.142	*w* = 1/[σ^2^(*F*_o_^2^) + (0.0617*P*)^2^ + 0.2511*P*] where *P* = (*F*_o_^2^ + 2*F*_c_^2^)/3
*S* = 1.05	(Δ/σ)_max_ < 0.001
3506 reflections	Δρ_max_ = 0.61 e Å^−^^3^
259 parameters	Δρ_min_ = −0.56 e Å^−^^3^
0 restraints	Extinction correction: *SHELXL97* (Sheldrick, 2008), Fc^*^=kFc[1+0.001xFc^2^λ^3^/sin(2θ)]^-1/4^
Primary atom site location: structure-invariant direct methods	Extinction coefficient: 0.014 (3)

### Special details

**Table:**

Geometry. All e.s.d.'s (except the e.s.d. in the dihedral angle between two l.s. planes) are estimated using the full covariance matrix. The cell e.s.d.'s are taken into account individually in the estimation of e.s.d.'s in distances, angles and torsion angles; correlations between e.s.d.'s in cell parameters are only used when they are defined by crystal symmetry. An approximate (isotropic) treatment of cell e.s.d.'s is used for estimating e.s.d.'s involving l.s. planes.
Refinement. Refinement of *F*^2^ against ALL reflections. The weighted *R*-factor *wR* and goodness of fit *S* are based on *F*^2^, conventional *R*-factors *R* are based on *F*, with *F* set to zero for negative *F*^2^. The threshold expression of *F*^2^ > σ(*F*^2^) is used only for calculating *R*-factors(gt) *etc*. and is not relevant to the choice of reflections for refinement. *R*-factors based on *F*^2^ are statistically about twice as large as those based on *F*, and *R*- factors based on ALL data will be even larger.

### Fractional atomic coordinates and isotropic or equivalent isotropic displacement parameters (Å^2^)

**Table:**

	*x*	*y*	*z*	*U*_iso_*/*U*_eq_	
Cl1	1.15532 (15)	0.75138 (11)	0.40321 (8)	0.0994 (4)	
O1	0.4993 (3)	1.13318 (17)	0.73705 (15)	0.0511 (5)	
N1	0.6371 (3)	0.9255 (2)	0.66884 (17)	0.0474 (6)	
N2	0.3133 (3)	1.10057 (19)	0.85631 (17)	0.0444 (5)	
N3	0.2491 (3)	1.0019 (2)	0.89847 (17)	0.0478 (6)	
C1	0.9987 (4)	0.7976 (3)	0.4798 (2)	0.0569 (8)	
C2	0.8521 (4)	0.8344 (3)	0.4374 (2)	0.0568 (8)	
H2	0.8337	0.8327	0.3673	0.068*	
C3	0.7319 (4)	0.8742 (3)	0.5007 (2)	0.0482 (7)	
H3	0.6313	0.8988	0.4725	0.058*	
C4	0.7596 (4)	0.8780 (2)	0.6053 (2)	0.0428 (6)	
C5	0.9092 (4)	0.8414 (3)	0.6470 (2)	0.0550 (7)	
H5	0.9299	0.8450	0.7177	0.066*	
C6	1.0273 (4)	0.7997 (3)	0.5837 (2)	0.0610 (8)	
H6	1.1262	0.7730	0.6109	0.073*	
C7	0.5574 (4)	0.8763 (2)	0.73575 (19)	0.0405 (6)	
C8	0.5736 (4)	0.7455 (2)	0.7374 (2)	0.0424 (6)	
C9	0.4800 (4)	0.6275 (3)	0.6453 (2)	0.0553 (8)	
H9	0.4062	0.6305	0.5846	0.066*	
C10	0.4969 (5)	0.5063 (3)	0.6442 (3)	0.0709 (10)	
H10	0.4334	0.4271	0.5828	0.085*	
C11	0.6068 (6)	0.5014 (3)	0.7330 (3)	0.0746 (10)	
H11	0.6195	0.4193	0.7313	0.090*	
C12	0.6985 (5)	0.6180 (3)	0.8250 (3)	0.0647 (9)	
H12	0.7723	0.6145	0.8855	0.078*	
C13	0.6809 (4)	0.7401 (3)	0.8275 (2)	0.0517 (7)	
H13	0.7413	0.8185	0.8899	0.062*	
C14	0.4532 (4)	0.9448 (2)	0.79664 (19)	0.0398 (6)	
C15	0.4324 (4)	1.0689 (2)	0.7907 (2)	0.0413 (6)	
C16	0.3330 (4)	0.9113 (2)	0.8635 (2)	0.0433 (6)	
C17	0.2877 (5)	0.7911 (3)	0.8946 (3)	0.0597 (8)	
H17A	0.1911	0.7943	0.9342	0.090*	
H17B	0.4027	0.7927	0.9392	0.090*	
H17C	0.2404	0.7092	0.8302	0.090*	
C18	0.2310 (4)	1.2054 (2)	0.8705 (2)	0.0431 (6)	
C19	0.3398 (5)	1.3293 (3)	0.8740 (2)	0.0571 (8)	
H19	0.4674	1.3452	0.8704	0.069*	
C20	0.2567 (5)	1.4296 (3)	0.8829 (3)	0.0682 (9)	
H20	0.3288	1.5130	0.8845	0.082*	
C21	0.0701 (5)	1.4074 (3)	0.8896 (2)	0.0669 (9)	
H21	0.0147	1.4747	0.8944	0.080*	
C22	−0.0349 (5)	1.2852 (3)	0.8891 (2)	0.0640 (9)	
H22	−0.1609	1.2709	0.8953	0.077*	
C23	0.0437 (4)	1.1834 (3)	0.8794 (2)	0.0554 (7)	
H23	−0.0286	1.1008	0.8790	0.067*	
H1	0.605 (4)	1.002 (3)	0.669 (2)	0.067 (9)*	

### Atomic displacement parameters (Å^2^)

**Table:**

	*U*^11^	*U*^22^	*U*^33^	*U*^12^	*U*^13^	*U*^23^
Cl1	0.0867 (7)	0.1399 (9)	0.0781 (6)	0.0596 (6)	0.0465 (5)	0.0223 (6)
O1	0.0581 (13)	0.0564 (11)	0.0614 (12)	0.0307 (9)	0.0305 (10)	0.0351 (9)
N1	0.0555 (15)	0.0529 (13)	0.0502 (13)	0.0312 (11)	0.0243 (11)	0.0253 (11)
N2	0.0529 (14)	0.0436 (12)	0.0490 (12)	0.0244 (10)	0.0232 (11)	0.0225 (10)
N3	0.0531 (15)	0.0520 (13)	0.0505 (13)	0.0245 (11)	0.0226 (11)	0.0254 (11)
C1	0.0485 (18)	0.0643 (18)	0.0495 (17)	0.0193 (14)	0.0190 (14)	0.0085 (14)
C2	0.057 (2)	0.0678 (18)	0.0444 (15)	0.0203 (15)	0.0179 (14)	0.0192 (14)
C3	0.0483 (17)	0.0539 (16)	0.0490 (16)	0.0197 (13)	0.0147 (13)	0.0251 (13)
C4	0.0400 (16)	0.0441 (14)	0.0451 (14)	0.0153 (12)	0.0151 (12)	0.0153 (12)
C5	0.0515 (18)	0.0749 (19)	0.0425 (15)	0.0307 (15)	0.0135 (13)	0.0197 (14)
C6	0.0484 (18)	0.079 (2)	0.0564 (18)	0.0333 (15)	0.0126 (14)	0.0184 (15)
C7	0.0394 (15)	0.0438 (14)	0.0387 (13)	0.0177 (11)	0.0065 (12)	0.0152 (11)
C8	0.0470 (17)	0.0422 (14)	0.0445 (15)	0.0221 (12)	0.0190 (13)	0.0163 (12)
C9	0.065 (2)	0.0500 (16)	0.0475 (16)	0.0203 (14)	0.0168 (14)	0.0126 (13)
C10	0.101 (3)	0.0409 (17)	0.065 (2)	0.0197 (17)	0.035 (2)	0.0109 (15)
C11	0.115 (3)	0.0557 (19)	0.090 (3)	0.051 (2)	0.064 (2)	0.0407 (19)
C12	0.081 (2)	0.075 (2)	0.070 (2)	0.0506 (18)	0.0323 (18)	0.0418 (18)
C13	0.0607 (19)	0.0509 (16)	0.0500 (16)	0.0285 (14)	0.0162 (14)	0.0193 (13)
C14	0.0420 (16)	0.0412 (14)	0.0412 (13)	0.0183 (11)	0.0123 (12)	0.0180 (11)
C15	0.0409 (16)	0.0458 (14)	0.0432 (14)	0.0199 (12)	0.0134 (12)	0.0192 (12)
C16	0.0442 (16)	0.0451 (14)	0.0437 (14)	0.0185 (12)	0.0134 (12)	0.0172 (12)
C17	0.068 (2)	0.0565 (17)	0.074 (2)	0.0273 (15)	0.0325 (16)	0.0370 (15)
C18	0.0486 (17)	0.0429 (14)	0.0420 (14)	0.0238 (12)	0.0143 (12)	0.0140 (11)
C19	0.063 (2)	0.0514 (17)	0.071 (2)	0.0299 (15)	0.0297 (16)	0.0271 (15)
C20	0.090 (3)	0.0549 (18)	0.084 (2)	0.0412 (18)	0.044 (2)	0.0351 (16)
C21	0.085 (3)	0.069 (2)	0.065 (2)	0.0523 (19)	0.0249 (18)	0.0240 (16)
C22	0.052 (2)	0.072 (2)	0.0669 (19)	0.0360 (17)	0.0168 (15)	0.0136 (16)
C23	0.0482 (18)	0.0525 (16)	0.0617 (18)	0.0201 (13)	0.0164 (14)	0.0139 (14)

### Geometric parameters (Å, °)

**Table:**

Cl1—C1	1.737 (3)	C10—C11	1.370 (5)
O1—C15	1.244 (3)	C10—H10	0.9300
N1—C7	1.337 (3)	C11—C12	1.379 (5)
N1—C4	1.426 (3)	C11—H11	0.9300
N1—H1	0.93 (3)	C12—C13	1.381 (4)
N2—C15	1.374 (3)	C12—H12	0.9300
N2—N3	1.403 (3)	C13—H13	0.9300
N2—C18	1.419 (3)	C14—C16	1.431 (4)
N3—C16	1.311 (3)	C14—C15	1.448 (3)
C1—C2	1.369 (4)	C16—C17	1.496 (4)
C1—C6	1.379 (4)	C17—H17A	0.9600
C2—C3	1.383 (4)	C17—H17B	0.9600
C2—H2	0.9300	C17—H17C	0.9600
C3—C4	1.382 (4)	C18—C19	1.380 (4)
C3—H3	0.9300	C18—C23	1.380 (4)
C4—C5	1.382 (4)	C19—C20	1.386 (4)
C5—C6	1.377 (4)	C19—H19	0.9300
C5—H5	0.9300	C20—C21	1.366 (5)
C6—H6	0.9300	C20—H20	0.9300
C7—C14	1.393 (3)	C21—C22	1.372 (4)
C7—C8	1.491 (3)	C21—H21	0.9300
C8—C13	1.378 (4)	C22—C23	1.377 (4)
C8—C9	1.389 (4)	C22—H22	0.9300
C9—C10	1.376 (4)	C23—H23	0.9300
C9—H9	0.9300		
			
C7—N1—C4	128.4 (2)	C11—C12—C13	120.0 (3)
C7—N1—H1	110.4 (18)	C11—C12—H12	120.0
C4—N1—H1	121.2 (18)	C13—C12—H12	120.0
C15—N2—N3	112.28 (19)	C8—C13—C12	119.9 (3)
C15—N2—C18	127.5 (2)	C8—C13—H13	120.0
N3—N2—C18	119.5 (2)	C12—C13—H13	120.0
C16—N3—N2	106.1 (2)	C7—C14—C16	132.6 (2)
C2—C1—C6	121.1 (3)	C7—C14—C15	121.7 (2)
C2—C1—Cl1	119.7 (2)	C16—C14—C15	105.3 (2)
C6—C1—Cl1	119.2 (2)	O1—C15—N2	126.0 (2)
C1—C2—C3	118.9 (3)	O1—C15—C14	129.5 (2)
C1—C2—H2	120.6	N2—C15—C14	104.4 (2)
C3—C2—H2	120.6	N3—C16—C14	111.7 (2)
C4—C3—C2	120.8 (3)	N3—C16—C17	118.0 (2)
C4—C3—H3	119.6	C14—C16—C17	130.3 (2)
C2—C3—H3	119.6	C16—C17—H17A	109.5
C5—C4—C3	119.6 (2)	C16—C17—H17B	109.5
C5—C4—N1	121.8 (2)	H17A—C17—H17B	109.5
C3—C4—N1	118.6 (2)	C16—C17—H17C	109.5
C6—C5—C4	119.8 (3)	H17A—C17—H17C	109.5
C6—C5—H5	120.1	H17B—C17—H17C	109.5
C4—C5—H5	120.1	C19—C18—C23	120.3 (2)
C5—C6—C1	119.9 (3)	C19—C18—N2	119.2 (2)
C5—C6—H6	120.1	C23—C18—N2	120.5 (2)
C1—C6—H6	120.1	C18—C19—C20	119.2 (3)
N1—C7—C14	118.9 (2)	C18—C19—H19	120.4
N1—C7—C8	118.6 (2)	C20—C19—H19	120.4
C14—C7—C8	122.4 (2)	C21—C20—C19	120.7 (3)
C13—C8—C9	119.8 (2)	C21—C20—H20	119.7
C13—C8—C7	121.4 (2)	C19—C20—H20	119.7
C9—C8—C7	118.8 (2)	C20—C21—C22	119.6 (3)
C10—C9—C8	119.7 (3)	C20—C21—H21	120.2
C10—C9—H9	120.2	C22—C21—H21	120.2
C8—C9—H9	120.2	C21—C22—C23	120.9 (3)
C11—C10—C9	120.5 (3)	C21—C22—H22	119.5
C11—C10—H10	119.8	C23—C22—H22	119.5
C9—C10—H10	119.8	C22—C23—C18	119.3 (3)
C10—C11—C12	120.0 (3)	C22—C23—H23	120.4
C10—C11—H11	120.0	C18—C23—H23	120.4
C12—C11—H11	120.0		

### Hydrogen-bond geometry (Å, °)

**Table:**

*D*—H···*A*	*D*—H	H···*A*	*D*···*A*	*D*—H···*A*
N1—H1···O1	0.93 (3)	1.87 (3)	2.701 (3)	146 (2)
